# 顶空固相微萃取-气相色谱-质谱法快速筛查油性基质中化学武器公约相关化合物

**DOI:** 10.3724/SP.J.1123.2022.07007

**Published:** 2023-04-08

**Authors:** Jia CHEN, Yulong LIU, Bin XU, Qin LIU, Jianwei XIE

**Affiliations:** 军事医学研究院毒物药物研究所, 抗毒药物与毒理学国家重点实验室, 北京 100850; State Key Laboratory of Toxicology and Medical Countermeasures, Institute of Pharmacology and Toxicology, Academy of Military Medical Sciences, Beijing 100850, China

**Keywords:** 顶空固相微萃取, 气相色谱-质谱联用, 化学武器公约相关化合物, 油性基质, 快速筛查, headspace solid-phase microextraction (HS-SPME), gas chromatography-mass spectrometry (GC-MS), Chemical Weapon Convention (CWC)-related chemicals, oil matrix, rapid screening

## Abstract

《化学武器公约》核查的化合物范围广泛,数量庞大,而核查样品通常来源复杂多样,且目标化合物含量很低,极易发生漏检和误检。鉴于核查结果的政治、军事敏感性,建立快速、有效的筛查方法对于准确鉴定复杂环境样品中公约相关化合物十分重要。针对油性基质样品前处理费时费力,低沸点化合物易漏检等问题,研究建立了顶空固相微萃取(HS-SPME)结合全扫描模式下气相色谱-质谱(GC-MS)检测,快速筛查油性基质中化学武器公约相关化合物的分析方法。为模拟筛查过程,选取24种性质各异的重要公约清单物为代表性化合物,并根据性质将所选化合物分为3组分别进行研究。对HS-SPME的工作条件进行了优化,包括萃取纤维涂层种类、萃取温度、萃取时间、解吸时间、衍生化方法等。对极性较低的挥发性和半挥发性公约相关化合物,采用二乙烯基苯/碳分子筛/聚二甲基硅氧烷(DVB/CAR/PDMS)纤维提取,以分流模式(分流比10∶1)进样分析,检出限为0.5~100 ng/mL。对中等极性和较高极性化合物,衍生化后采用聚二甲基硅氧烷/二乙烯基苯(PDMS/DVB)纤维提取,以不分流模式进样分析,检出限为20~300 ng/mL。所建方法简单、快速、灵敏,特别是可避免样品浓缩导致的低沸点化合物的损失和漏检,可用于复杂油性基质中痕量化学武器公约相关化合物的快速筛查与鉴定,并已多次成功应用于国际禁止化学武器组织效能水平测试中。

## Abstract (en)

The Chemical Weapons Convention (CWC) requires verification of a large number of compounds with different types and properties. The results of the verification are of great political and military sensitivity. However, the sources of verification samples are complex and diverse, and the contents of the target compounds in these samples are usually very low. These issues increase the likelihood of missed or false detection. Thus, establishing rapid and effective screening methods for the accurate identification of CWC-related compounds in complex environmental samples are of great importance. In this study, a fast and simple procedure based on headspace solid-phase microextraction (HS-SPME) followed by gas chromatography-electron ionization mass spectrometry (GC-EI/MS) in full-scan mode was developed to determine CWC-related chemicals in oil matrix. A total of 24 CWC-related chemicals with different chemical characteristics were selected to simulate the screening procedure. The selected compounds were divided into three groups based on their properties. The first group included volatile and semi-volatile CWC-related compounds with relatively low polarity, which could be extracted by HS-SPME and directly analyzed by GC-MS. The second group included moderately polar compounds with hydroxyl or amino groups; such compounds are related to nerve, blister, and incapacitating agents. The compounds in the third group included non-volatile CWC-related chemicals with relatively strong polarity, such as alkyl methylphosphonic acids and diphenyl hydroxyacetic acid. These compounds must be derivatized into vaporizable derivatives prior to extraction by HS-SPME and analysis by GC-MS. Variables that influence the SPME process, such as fiber type, extraction temperature and time, desorption time, and derivatization protocol, were optimized to improve the sensitivity of the method. The screening procedure for CWC-related compounds in the oil matrix samples included two main steps. First, low-polarity volatile and semi-volatile compounds (i. e. the first group) were extracted by HS-SPME with divinylbenzene/carboxen/polydimethylsiloxane (DVB/CAR/PDMS) fibers and analyzed in split-injection mode (split ratio, 10∶1) using GC-MS. The use of a large split ratio can reduce the solvent effect, which is conducive to the detection of low-boiling-point compounds. If necessary, the sample could be extracted once more and analyzed in splitless mode. The derivatization agent bis(trimethylsilyl)trifluoroacetamide (BSTFA) was then added to the sample. Mid- and high-polarity compounds (i. e. the second and third groups) were extracted with polydimethylsiloxane/divinylbenzene (PDMS/DVB) fibers after derivatization and analyzed in splitless mode using GC-MS. The established method exhibited good repeatability and sensitivity. The detection limits for the compounds in the first group ranged from 0.5 ng/mL to 100 ng/mL, whereas the detection limits for the compounds in the second and third groups ranged from 20 ng/mL to 300 ng/mL. Except for compounds with extremely high boiling points and a few compounds that are not suitable for derivatization with BSTFA, the method can be used to analyze most CWC-related compounds in oil matrix samples. In particular, it greatly shortened the preparation time of the oil matrix samples and reduced the loss of low-boiling-point compounds owing to the sample concentration process, thereby avoiding missed detection. The method was successfully applied to the Organization for the Prohibition of Chemical Weapons (OPCW) proficiency tests and proved to be a useful technique for the rapid screening of trace levels of CWC-related chemicals in oil matrix.

对化学武器公约相关化合物的分析检测是《国际禁止化学武器公约》(以下简称《公约》)规定的核查关键技术之一,检测结果将作为缔约国是否履约的直接证据,具有重要的政治、军事和外交意义。《公约》核查的化合物范围很广,除《公约》附表中所规定的47大类化学品外,还包括它们的氧化产物、还原产物、降解产物等(统称“公约相关化合物”),数量庞大,且性质各异。而从化学武器使用现场及核查设施中采集的样品通常来源复杂多样,且目标化合物含量很低,极易发生漏检和误检。因此建立全面、快速、有效的样品筛查方法对于准确鉴定复杂环境样品中公约相关化合物十分重要。

油性基质样品,如机油、泵油、生物柴油、食用油等是核查现场可能采集到的一类较为特殊也较难处理的有机样品。由于直接采用气相色谱-质谱(GC-MS)方法分析时会污染仪器,并产生严重背景干扰,影响低浓度目标化合物的检测,此类样品通常应采用国际禁止化学武器公约组织(OPCW)发布的《化武相关分析推荐操作规程》(ROP)^[1]^中推荐的溶剂交换法^[1,2]^处理后再进行分析。但该方法不仅费时费力(>5 h),一些低沸点目标化合物还极易在最后的样品浓缩过程中损失而导致漏检。这也是在OPCW每年举行的环境样品效能水平测试中此类样品的检出率普遍偏低的主要原因,例如在OPCW第42次水平测试中,参试实验室对泵油样品中芥子气相关化合物二乙烯基硫醚的检出率仅为50%。

固相微萃取(SPME)是集萃取、浓缩和进样于一体的多功能样品前处理技术,具有操作简便快速、样品用量少等优点^[3]^。将SPME用于公约相关化合物的分析已有文献报道^[4⇓⇓⇓⇓⇓⇓-11]^,但通常分析对象都是特定类型的化合物,如神经性毒剂原形及水解产物^[4,5]^、胺吸膦^[6]^、芥子气相关化合物^[8,11]^、路易氏剂水解及氧化产物^[9,11]^、氰化物^[10]^等,涉及的样品基质类型主要是水样^[4,6,11]^、土壤样品^[7,11]^、空气样品^[4]^、植物^[6]^、织物^[5]^和生物样品^[10]^等。顶空固相微萃取(HS-SPME)由于受基质干扰小,非常适合分析油性基质中的挥发性化合物^[12⇓-14]^,但将HS-SPME用于油性基质中公约相关化合物分析目前国内外尚未见报道。近年来,将SPME用于化合物的非靶向筛查屡有报道^[15,16]^,本研究将顶空固相微萃取-气相色谱-质谱联用(HS-SPME-GC-MS)技术用于复杂油性基质中公约相关化合物的筛查与鉴定,以油性基质中极性较低的挥发性和半挥发性公约相关化合物(尤其是最易漏检的低沸点挥发性公约相关化合物)为重点关注对象,建立了HS-SPME-GC-MS分析方法;同时针对中等极性和强极性公约相关化合物建立了衍生化HS-SPME-GC-MS分析方法。所建立的方法可比较全面、快速地筛查油性基质中的公约相关化合物,在低沸点化合物筛查方面尤具优势,可作为推荐方法的补充方法进一步确保检测结果的准确性,并已多次成功应用于OPCW效能水平测试中。

## 1 实验部分

### 1.1 仪器与试剂

Agilent 6890N-5975B气相色谱-质谱联用仪(美国Agilent公司); PC-420D磁力加热搅拌器(美国Corning公司);固相微萃取装置采样台(北京康林科技有限公司); SPME手柄、不同涂层SPME萃取纤维头: 65 μm聚二甲基硅氧烷/二乙烯基苯(PDMS/DVB)、50/30 μm二乙烯基苯/碳分子筛/聚二甲基硅氧烷(DVB/CAR/PDMS)、75 μm碳分子筛/聚二甲基硅氧烷(CAR/PDMS)、85 μm聚丙烯酸酯(PA)、100 μm聚二甲基硅氧烷(PDMS)、65 μm聚乙二醇/二乙烯基苯(CW/DVB)、60 μm聚乙二醇(PEG)以及4 mL顶空样品瓶均购自美国Supelco公司。所有SPME萃取纤维头在使用前均按照生产厂家所提供的条件进行了活化处理。

片呐醇(纯度99%)购自Alfa Aesar;亚膦酸二甲酯(纯度98%)、1,4-噻烷(纯度98%)、1,4-二噻烷(纯度97%)、硫二苷醇(纯度98%)和甲基膦酸二乙酯(纯度97%)购自Aldrich;喹咛醇(纯度95%)、甲基二乙醇胺(纯度97%)、甲基二乙醇胺(纯度96%)、三乙醇胺(纯度97%)、甲基膦酸(纯度98%)、乙基甲基膦酸(纯度97%)、异丙基甲基膦酸(纯度95%)、异丁基甲基膦酸(纯度98%)、片呐基甲基膦酸(纯度97%)、环己基甲基膦酸(纯度97%)和二苯羟乙酸(纯度98%)购自Sigma;氯化苦(纯度95%)、沙林(纯度97%)和芥子气(纯度96%)由防化学院提供;甲基膦酸甲基环戊基酯(纯度>95%)、2-甲基-1,3,2-二硫代膦-2-硫化物(纯度>95%)、甲基乙基乙醇胺(纯度>95%)、甲基丙基乙醇胺(纯度>95%)由本实验室自行合成。真空泵油(Edwards 45)购自Edwards;二氯甲烷、乙腈和正己烷(HPLC级)购自Duksan;双三甲基硅基三氟乙酰胺(BSTFA)(GC级)购自Merck;实验用水均为Milli Q超纯水。

### 1.2 溶液配制

将待考察化合物分别以二氯甲烷、正己烷或乙腈为溶剂配制成质量浓度约5 mg/mL的储备液,置于4 ℃冰箱保存。向泵油中添加各化合物的储备液,使泵油溶液中各化合物的质量浓度均为5 μg/mL,以此溶液作为优化HS-SPME条件的工作溶液。工作溶液每周新鲜配制,以保证其中化合物的稳定性。用泵油对工作溶液进行逐级稀释,考察方法的重复性和灵敏度。

### 1.3 GC-MS条件

GC条件 采用HP-5ms石英毛细管色谱柱(25 m×0.2 mm×0.33 μm,美国J&W Scientific公司);载气为氦气,流速0.8 mL/min;进样口温度250 ℃(使用CW/DVB和PEG纤维头时,进样口温度为220 ℃);升温程序如下:35 ℃保持3 min,以15 ℃/min升至290 ℃,保持3 min。分流方式:HS-SPME采用10∶1分流进样,衍生化HS-SPME采用0.5 min不分流进样。

MS条件 电离方式:电子轰击电离(EI, 70 eV);离子源温度230 ℃;四极杆温度150 ℃;溶剂延迟3 min(HS-SPME)/7.5 min(衍生化HS-SPME);采用全扫描(SCAN)检测模式,质谱扫描范围*m/z* 40~550;由Chemstation工作软件采集的数据经AMDIS(质谱自动去卷积与识别系统)软件进行去卷积处理后,使用最新版的OCAD(OPCW中心数据库)和NIST2015数据库对检测到的化合物进行谱库检索和比对。

### 1.4 样品前处理

#### 1.4.1 HS-SPME方法

取1 mL泵油样品置于4 mL顶空样品瓶中,密封后于70 ℃下以600 r/min磁力加热搅拌20 min。插入DVB/CAR/PDMS萃取纤维头顶空萃取30 min。萃取完毕,将萃取纤维插入气相色谱进样口,解吸5 min后进行GC-MS分析。

#### 1.4.2 衍生化HS-SPME方法

取1 mL泵油样品置于4 mL顶空样品瓶中,加入10 μL BSTFA,密封后于80 ℃下以600 r/min磁力加热搅拌30 min。插入PDMS/DVB萃取纤维头顶空萃取30 min。萃取完毕,将萃取纤维插入气相色谱进样口,解吸5 min后进行GC-MS分析。

#### 1.4.3 OPCW推荐处理方法(ROP)^[1,2]^

取1 mL泵油样品置于10 mL玻璃离心试管中,用2 mL正己烷稀释。向稀释后的样品中加入2 mL乙腈,涡旋提取15 min,然后以3000 r/min离心5 min使溶液分层,将上层乙腈层转移至另一10 mL玻璃离心试管中。剩余正己烷层再加入2 mL乙腈重复提取一次,合并两次提取液。向乙腈提取液中加入1 mL正己烷,涡旋混合15 min,将混合溶液密封,置于-20 ℃冰箱中静置2 h(注:此步骤用于去除乙腈提取液中带入的油性基质)。将上层乙腈层(约4 mL)转移至另一玻璃离心试管中,在温和氮气流下浓缩至约200 μL,直接或衍生化后进行GC-MS分析(进样量1 μL)。

## 2 结果与讨论

### 2.1 代表性公约相关化合物的选择

本研究的主要目的不仅是建立一种简单、快速的样品前处理方法,还要重点解决油性基质中易挥发化合物的易漏检问题。根据化合物的性质,选择了24种具有代表性的公约相关化合物作为研究考察对象(见表1),并根据性质分成3组。第一组(化合物1~10),是极性相对较低、具有挥发性、可用HS-SPME方法萃取,并直接采用GC-MS方法分析的公约相关化合物。为了重点解决低沸点化合物易漏检的问题,选择了一些沸点低、极易挥发的公约相关化合物,如片呐醇、氯化苦、亚膦酸二甲酯等;同时为了兼顾方法的普适性,又选择了一些不同类型化学武器及其相关化合物,如神经性毒剂沙林和糜烂性毒剂芥子气,以及一些不同沸点的神经性毒剂和芥子气相关化合物。第二组(化合物11~17)是具有中等极性的醇类、胺类化合物,均为神经性毒剂、糜烂性毒剂或失能剂相关化合物,这类化合物通常具有一定的挥发性,也可直接用顶空方法萃取并用GC-MS检测,但其色谱行为受极性影响可能导致检测灵敏度较低,需采用衍生化的方法来提高检测灵敏度。第三组(化合物18~24)是具有较强极性的、必须先经过衍生化才能用顶空法进行萃取的难挥发性公约相关化合物,主要包括神经性毒剂的降解产物烷基膦酸,以及失能性毒剂毕兹(BZ)的降解产物二苯羟乙酸等。后两组主要考察了衍生化对提取和分析的影响。

**表1:** 所选择的代表性公约相关化合物

Group			No.	Compound		Structure	CWC schedule	
Ⅰ			1	pinacolyl alcohol (片呐醇)		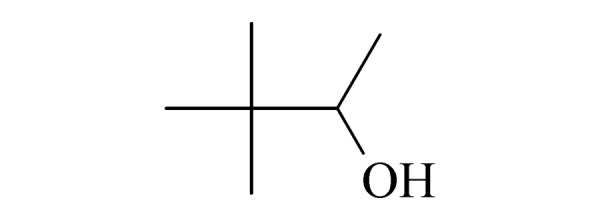	2.B.14	
			2	chloropicrin (氯化苦)		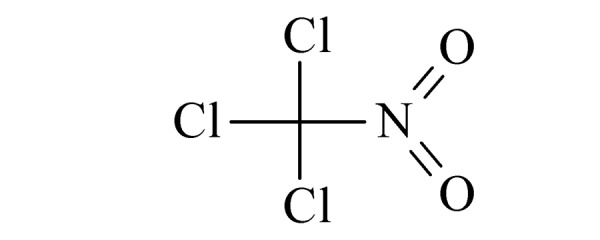	3.A.04	
			3	dimethyl phosphite (DMP) (亚膦酸二甲酯)		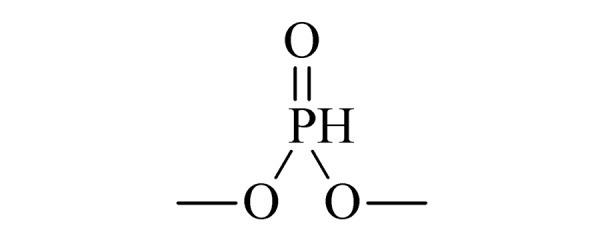	3.B.10	
			4	sarin (GB) (沙林)		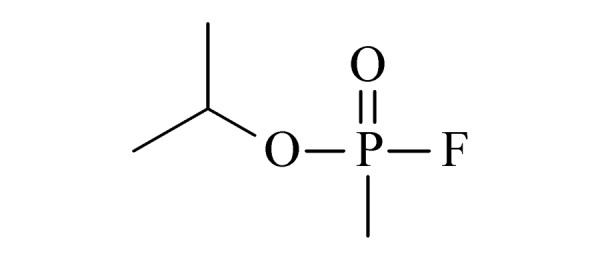	1.A.01	
			5	1,4-oxathiane (1,4-噻烷)		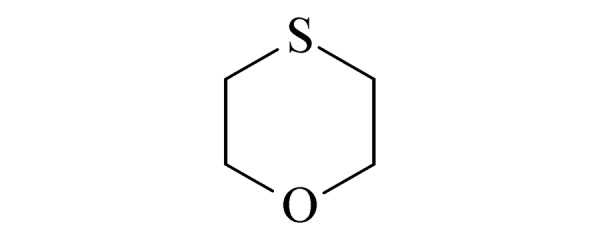	N.S.^*^	
			6	diethyl methylphosphonate (DEMP) (甲基膦酸二乙酯)		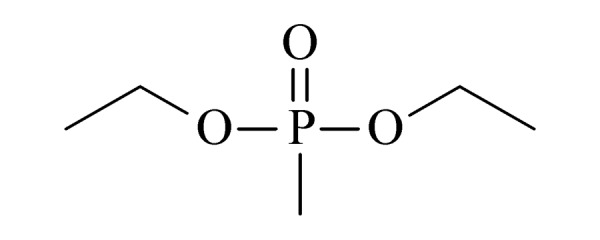	2.B.04	
Group	No.	Compound			Structure			CWC schedule
	7	1,4-dithiane (1,4-二噻烷)			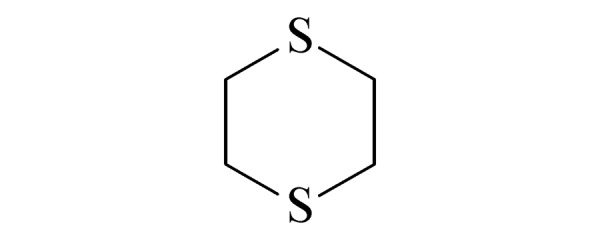			N.S.^*^
	8	sulfur mustard (SM) (芥子气)			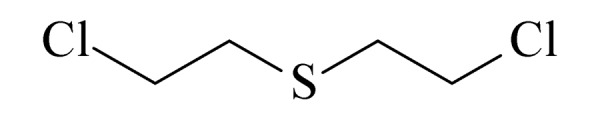			1.A.04
	9	cyclopentyl methyl methylphosphonate (CPMMP) (甲基膦酸甲基环戊基酯)			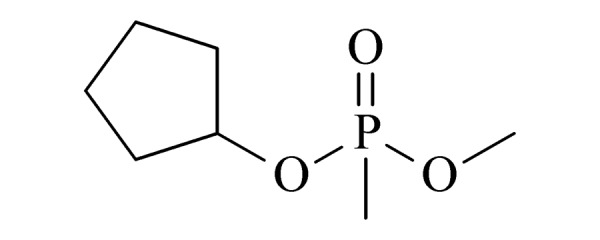			2.B.04
	10	2-methyl-1,3,2-dithiaphosphinane 2-sulfide (2-甲基-1,3,2-二硫代膦-2-硫化物)			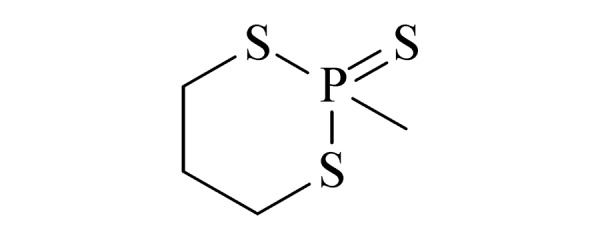			2.B.04
Ⅱ	11	N-ethyl N-methylaminoethanol (EMAE) (甲基乙基乙醇胺)			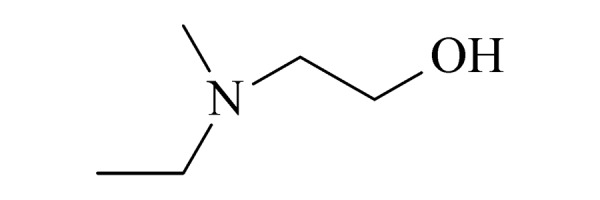			2.B.11
	12	N-methyl N-propylaminoethanol (MPAE) (甲基丙基乙醇胺)			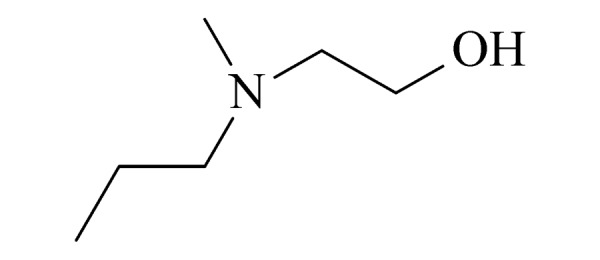			2.B.11
	13	methyldiethanolamine (MDEA) (甲基二乙醇胺)			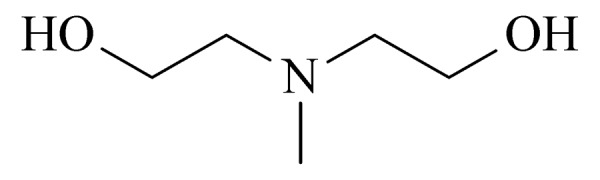			3.B.15
	14	ethyldiethanolamine (EDEA) (乙基二乙醇胺)			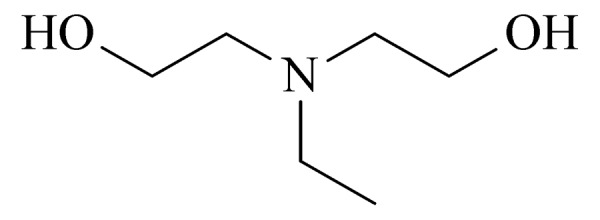			3.B.15
	15	triethanolamine (TEA) (三乙醇胺)			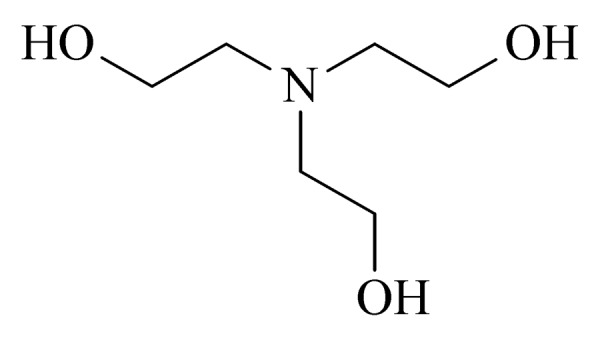			3.B.17
	16	thiodiglycol (TDG) (硫二苷醇)			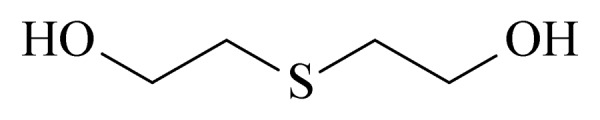			2.B.13
	17	quinuclidin-3-ol(喹咛醇)			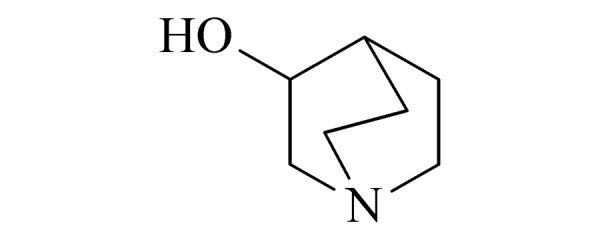			2.B.09
Ⅲ	18	methylphosphonic acid (MPA) (甲基膦酸)			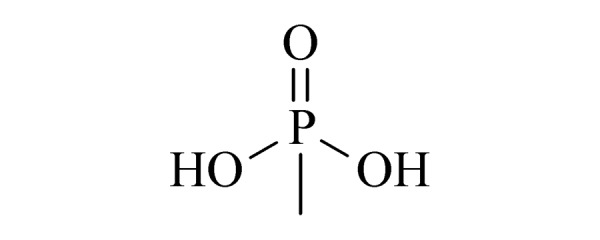			2.B.04
	19	ethyl methylphosphonic acid (EMPA) (乙基甲基膦酸)			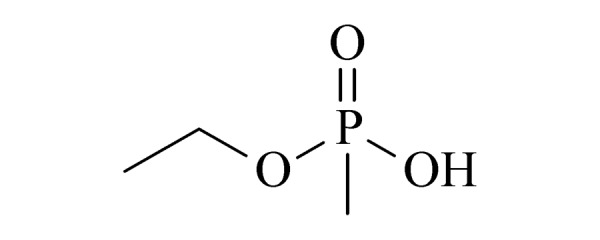			2.B.04
	20	isopropyl methylphosphonic acid (IMPA) (异丙基甲基膦酸)			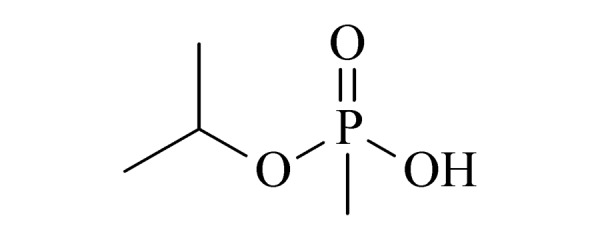			2.B.04
	21	isobutyl methylphosphonic acid (i-BuMPA) (异丁基甲基膦酸)			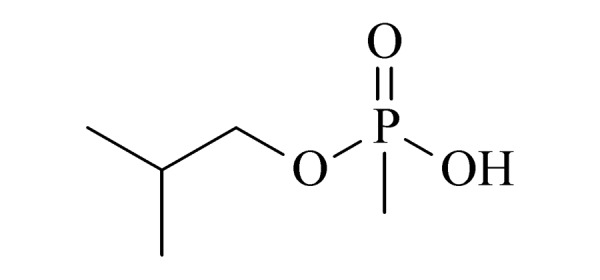			2.B.04
	22	pinacolyl methylphosphonic acid (PMPA) (片呐基甲基膦酸)			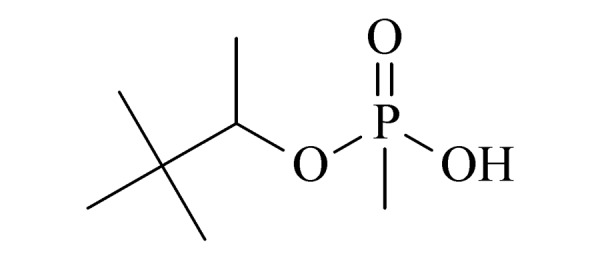			2.B.04
	23	cyclohexyl methylphosphonic acid (CHMPA) (环己基甲基膦酸)			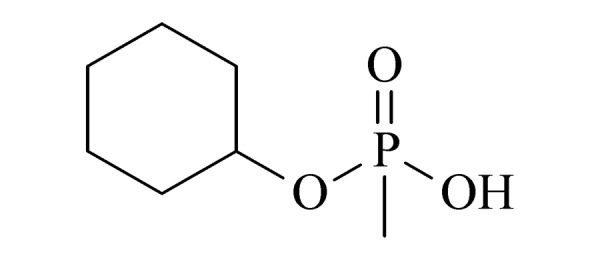			2.B.04
	24	2,2-diphenyl-2-hydroxyacetic acid (BZA) (二苯羟乙酸)			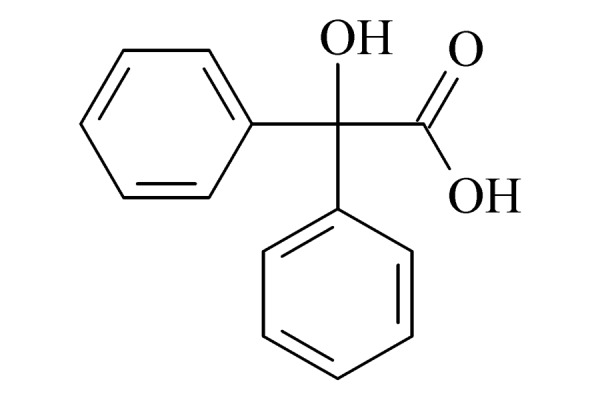			2.B.08

* N.S.: the compound is not listed in the CWC schedules but is the related chemical that should be reported.

### 2.2 GC-MS分析条件确定

对于HS-SPME-GC-MS分析,考虑到第一组分析对象中涉及片呐醇、氯化苦这一类低沸点化合物,为了有利于这类化合物和一些低沸点溶剂的分离,需设置较低的气相色谱起始温度,考察了35、40和50 ℃,最终确定升温程序的起始温度为35 ℃。另外,受溶剂效应的影响,低沸点化合物应采用较大的分流比才能获得较好的色谱峰形。考察了不分流及分流比为2∶1、5∶1、10∶1、20∶1时对于低沸点化合物峰形的影响。结果发现,采用SPME常用的不分流模式进样时,出峰时间靠前的低沸点公约相关化合物片呐醇、氯化苦、亚膦酸二甲酯等峰形严重展宽,影响检出。随着分流比增加,低沸点化合物峰形明显改善。此外,大分流比还有利于低沸点目标化合物与低沸点干扰溶剂的分离。为了保证低沸点化合物的检出,同时兼顾其他化合物的检测灵敏度,最终确定筛查时分流进样的分流比为10∶1。对于沸点较高、出峰时间较晚的化合物,采用不分流进样模式可大幅提高检测灵敏度,实际检测时可对样品进行重复提取,并采用不分流模式再次进样分析。对于衍生化HS-SPME-GC-MS分析,为提高检测灵敏度采用不分流进样。

基于普筛的目的,本方法采用气相色谱-电子轰击电离质谱(GC-EI/MS)的全扫描模式进行检测。获得的全扫描数据经AMDIS软件的去卷积处理,可消除背景干扰,自动识别和抽取出单个化合物的质谱图,并在专用(如OCAD库、实验室自建数据库等)和通用(如NIST库等)质谱数据库中进行谱图检索和比对,从而快速识别和发现样品中的公约相关化合物。

### 2.3 HS-SPME萃取条件的优化

实际样品中常见的油性基质包括泵油、柴油、汽油、生物柴油、食用油等。本研究以泵油为基质,考察了萃取条件,如萃取纤维涂层、萃取温度、萃取时间、解吸温度等对第一组中10种化合物萃取效果的影响。所有实验数据均为两次测定的平均值。

#### 2.3.1 萃取纤维涂层的选择

虽然第一组中的化合物极性均相对较低,但各化合物的性质差别较大,为了选择一种普适性较好的纤维涂层,在萃取温度70 ℃、萃取时间30 min的条件下考察了几种纤维涂层(65 μm PDMS/DVB、50/30 μm DVB/CAR/PDMS、75 μm CAR/PDMS、85 μm PA、100 μm PDMS、65 μm CW/DVB、60 μm PEG)对各目标化合物的萃取效果(见图1)。结果表明,不同类型纤维涂层对于不同化合物的吸附效果差异很大。CAR/PDMS对1,4-噻烷和1,4-二噻烷的萃取效率远远高于其他几种涂层,提示其对于此类含硫的小分子化合物可能具有特异的吸附能力。对于2-甲基-1,3,2-二硫代膦-2-硫化物,CW/DVB的萃取效果最好,DVB/CAR/PDMS次之。总体而言,DVB/CAR/PDMS对10种目标化合物均展现出较好的萃取效果,其次是PDMS/DVB,而CAR/PDMS、PA、PDMS、CW/DVB和PA都只是对部分化合物展现出较好的萃取效果。这可能是因为DVB/CAR/PDMS和PDMS/DVB都属于双极性涂层,对于不同极性的化合物均有一定的适用性。而其中DVB/CAR/PDMS实际上是PDMS/DVB和CAR/PDMS两种涂层的结合体,因此拥有这两种涂层的吸附特性,适用范围更广泛。从筛查的普适角度综合考虑,选用DVB/CAR/PDMS作为第一组筛查的纤维涂层。

**图1:**
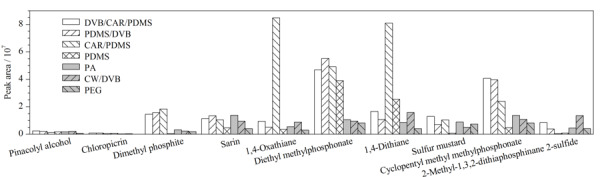
不同纤维涂层对所选择的公约相关化合物提取效果

#### 2.3.2 萃取温度的选择

温度是影响SPME萃取速度和效率的重要因素。对于HS-SPME,升温一方面有助于分析物从基质中挥发出来,提高检测灵敏度;另一方面,又会促使分析物从萃取涂层上脱附,降低萃取效率。鉴于温度对萃取的双重影响,在其他条件不变的情况下,考察了萃取温度为50、60、70、80、90 ℃时DVB/CAR/PDMS对各目标化合物萃取量的影响。实验结果表明(见图2),随着温度升高,低沸点化合物片呐醇、氯化苦、沙林等萃取效率均有所下降,中等沸点的1,4-二噻烷和芥子气呈现出随温度升高先增加后降低的趋势,而沸点较高的甲基膦酸甲基环戊基酯和2-甲基-1,3,2-二硫代膦-2-硫化物则随着温度升高,萃取效率明显增加。这可能是由于萃取过程中脱附作用对低沸点化合物的影响较大,而挥发度对较高沸点化合物的影响较大。此外,由于泵油样品本身也有一定的挥发性,当萃取温度达到80~90 ℃时,色谱图后段背景干扰明显增加。为了兼顾样品中的低沸点和较高沸点化合物的检测,选择70 ℃作为筛查的萃取温度。

**图2:**
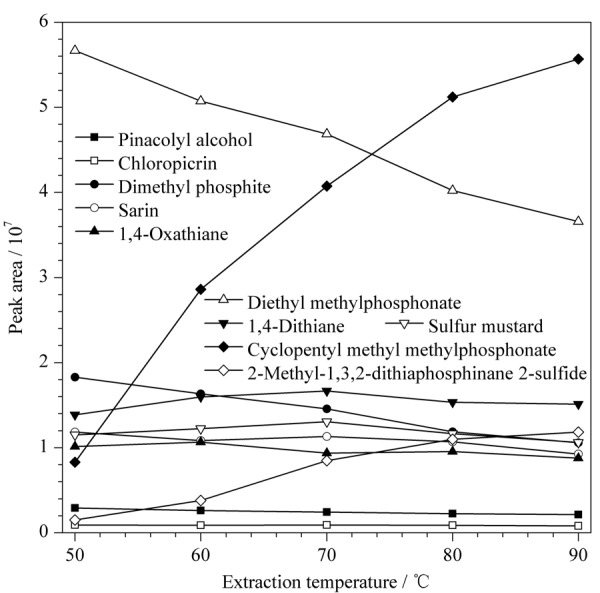
萃取温度对萃取效果的影响

#### 2.3.3 萃取时间的选择

在萃取温度70 ℃下,考察了萃取时间分别为10、20、30、40和60 min时DVB/CAR/PDMS对各目标化合物的萃取效果。结果(见图3)表明,对于挥发性较强、保留时间相对较短的化合物片呐醇、氯化苦、沙林、亚膦酸二甲酯、1,4-噻烷、1,4-二噻烷等,萃取10~20 min已基本达到平衡,且随着萃取时间增加,部分化合物呈现出萃取量逐渐减少的趋势。对于沸点较高、挥发性稍弱的化合物芥子气、甲基膦酸甲基环戊基酯和2-甲基-1,3,2-二硫代膦-2-硫化物则需要相对较长的平衡时间,随着萃取时间延长,萃取量逐渐增加,但30 min后增加趋势变缓。综合考虑样品处理时间和GC-MS分析周期,选择萃取时间为30 min。

**图3:**
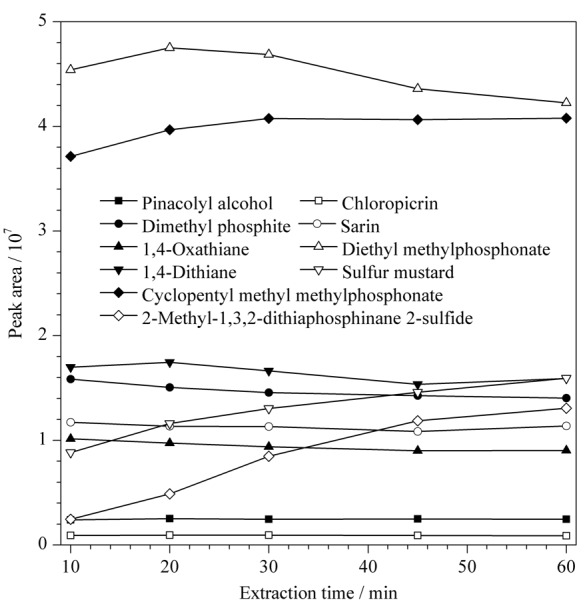
萃取时间对萃取效果的影响

#### 2.3.4 解吸条件的选择

通常提高解吸温度可以加快解吸速度,减小色谱的初始峰宽,但解吸温度不能高于纤维涂层的最高允许温度。DVB/CAR/PDMS纤维涂层的最高使用温度为270 ℃,因此将解吸温度设置为250 ℃,并在此温度下考察了解吸时间1、3、5 min对色谱峰响应值的影响,结果表明3 min之后可解吸完全,且二次解吸未见残留。为确保方法有更广泛的普适性,将解吸条件确定为250 ℃解吸5 min。

### 2.4 衍生化HS-SPME考察

SPME衍生化方法通常可分为3种,即样品基质中衍生化、萃取纤维上衍生化和GC进样口衍生化,其中前两种最为常用。第三组中的酸性化合物由于极性均较强,挥发性很低,只能采用基质中衍生化的方法,先转化为可挥发的衍生化产物,再使用顶空方法进行萃取分析。第二组的醇、胺类化合物由于本身有一定的挥发性,可以用HS-SPME方法直接进行提取分析。因此既可采用在基质中衍生化后进行顶空萃取的方法,也可采用萃取到纤维上再进行衍生化的方法进行分析。但羟基、氨基的存在导致这类化合物在非极性色谱柱上的色谱行为不佳,从而影响了检测灵敏度。实验表明,对这类化合物,特别是多羟基化合物如TDG、MDEA、EDEA和TEA等,进行衍生化后可大幅提高检测灵敏度。比较了两种衍生化方法对第二组醇、胺类化合物的分析效果,结果表明,除TEA外,其他几种化合物采用萃取纤维上衍生化的检测灵敏度略高于采用基质中衍生化的方法。TEA是第二组7个化合物中极性最强、挥发性最低的化合物,因此采用基质中衍生化方法的提取率远高于在萃取纤维上衍生化。综合考虑第二组和第三组化合物的分析情况,为简化实验过程,缩短分析周期,选择统一采用基质中衍生化的方法分析第二组和第三组中的化合物,并以较为通用的BSTFA作为衍生化试剂。

考察了衍生化试剂BSTFA用量对萃取效果的影响。在1 mL样品中分别加入5、10、20、50、100 μL BSTFA,结果表明,对于单衍生位点化合物,每mL样品中加入5 μL或10 μL BSTFA时,衍生化产物的检测灵敏度最高且相差不大,继续增加用量,峰高显著降低;这可能是由于大量的BSTFA产生竞争吸附,从而影响到衍生化产物的提取效率。对于多(双或三)衍生位点化合物,每mL样品中加入10 μL或20 μL BSTFA时,衍生化产物的检测灵敏度最高,提示适当增加衍生化试剂用量有利于提高过多衍生位点化合物的衍生化效率,最终确定衍生化试剂用量为每mL样品中加入10 μL BSTFA。

考察了不同纤维涂层(65 μm PDMS/DVB、50/30 μm DVB/CAR/PDMS、75 μm CAR/PDMS、100 μm PDMS)对各衍生化产物的提取效果,结果表明,PDMS/DVB和DVB/CAR/PDMS提取效果差不多,PDMS次之,CAR/PDMS提取效果较差。因此选择PDMS/DVB用于第二组和第三组化合物衍生化产物的筛查。

为了简化实验过程,在同一温度下对样品进行衍生化反应和萃取。根据以往经验,采用BSTFA为衍生化试剂时,大多数化合物在60 ℃下加热30 min即可获得较完全的衍生化。考察了在不同温度(60、70、80、90、100 ℃)下衍生化30 min后进行萃取的效果,结果表明,随着温度升高,沸点相对较低的衍生化产物如EMAE和MPAE的硅烷化产物信号会有所降低,而沸点较高的衍生化产物如BZA的硅烷化产物信号则显著提升,但温度升高后色谱图后段的背景干扰明显增加。综合考虑后将衍生化和萃取温度设置为80 ℃,萃取时间30 min。

### 2.5 灵敏度和重复性考察

由于本研究侧重于定性筛查分析,因此仅对方法的灵敏度和重复性进行了考察。在已优化的萃取条件下,对添加水平均为1 μg/mL的泵油样品平行检测6次,计算各化合物峰面积的重复性。结果显示,HS-SPME-GC-MS分析时各化合物峰面积的相对标准偏差(RSD)为1.7%~11.6%(见表2),衍生化HS-SPME-GC-MS分析时各化合物峰面积的RSD为3.9%~19.4%(见表3),方法的重复性可接受。

**表2:** HS-SPME-GC-MS分析的检出限和精密度(*n*=6)

Compound	t_R_/ min	Extracted ion (m/z)	LOD^*^/ (ng/mL)	RSD^**^/ %
Pinacolyl alcohol	4.61	57	5	4.3
Chloropicrin	5.46	117	100	11.6
Dimethyl phosphite	5.90	80	30	8.4
Sarin	6.14	99	1	3.9
1,4-Oxathiane	7.13	104	2	4.2
Diethyl	8.77	97	0.5	1.7
methylphosphonate				
1,4-Dithiane	9.52	120	1	3.3
Sulfur mustard	10.68	109	1	4.5
Cyclopentyl methyl	11.60	111	0.5	2.8
methylphosphonate				
2-Methyl-1,3,2-dithia-	15.28	184	30	7.7
phosphinane 2-sulfide				

*S/N≥5; ** at 1 μg/mL level.

**表3:** 衍生化HS-SPME-GC-MS分析方法的检出限和精密度(*n*=6)

Compound	t_R_/ min	Extracted ion (m/z)	LOD^*^/ (ng/mL)	RSD^**^/ %
EMAE (TMS)	8.14	72	50	8.5
MPAE (TMS)	9.06	86	50	7.3
MDEA (2TMS)	11.66	160	30	6.8
EDEA (2TMS)	12.13	174	30	3.9
TEA (3TMS)	14.45	262	100	10.7
TDG (2TMS)	12.70	116	20	8.3
Quinuclidin-3-ol (TMS)	11.28	200	20	14.3
MPA (2TMS)	10.04	225	50	6.7
EMPA (TMS)	9.34	153	20	4.6
IMPA (TMS)	9.64	153	20	5.9
i-BuMPA (TMS)	10.78	153	30	9.6
PMPA (TMS)	11.73	153	50	11.2
CHMPA (TMS)	13.27	153	50	8.8
BZA (2TMS)	16.47	255	300	19.4

*S/N≥5; ** at 1 μg/mL level. TMS: trimethylsilyl ester.

在全扫描监测模式下,以逐级稀释的方法考察了方法的灵敏度。以提取离子色谱图(EIC)中各化合物特征离子的5倍信噪比(*S/N*≥5)计算得到泵油样品中各化合物的检出限。第一组10种化合物大部分检出限为0.5~30 ng/mL,仅氯化苦检出限为100 ng/mL;第二组和第三组化合物的三甲基硅烷化衍生化产物的峰高随浓度降低急剧下降,影响了检测的灵敏度,其中沸点最高的BZA(2TMS)由于挥发度低,检出限仅为300 ng/mL,其余化合物检出限均为20~100 ng/mL。尽管化合物性质各异导致检测灵敏度存在显著差异,总体而言本方法的检测灵敏度已足够满足环境样品中公约相关化合物筛查的使用要求(注:OPCW对环境样品检测的要求为质量浓度大于1 μg/mL的公约相关化合物均需报告)。

采用HS-SPME-GC-MS分析泵油加标样品中第一组10种公约相关化合物(质量浓度均为1 μg/mL)的总离子流色谱图(TIC)见图4。

**图4:**
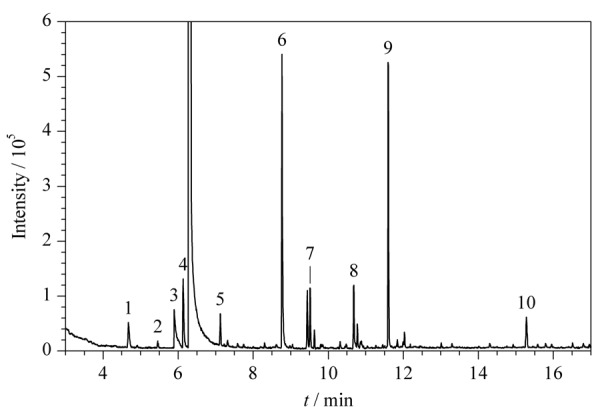
HS-SPME-GC-MS分析泵油加标样品中第一组10种公约相关化合物的总离子流图

采用衍生化HS-SPME-GC-MS分析泵油加标样品中第二组和第三组公约相关化合物(质量浓度均为1 μg/mL)的TIC图见图5。

**图5:**
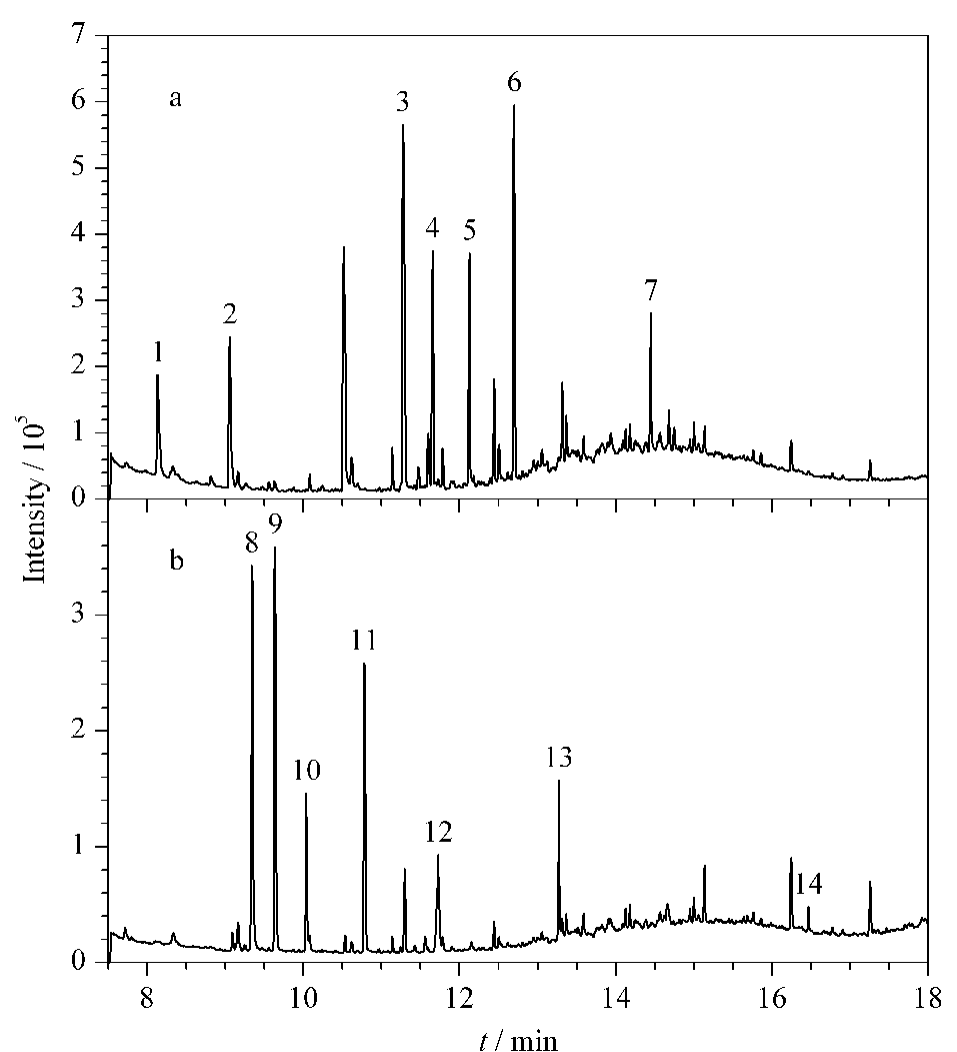
衍生化HS-SPME-GC-MS分析泵油加标样品中第二组和第三组公约相关化合物的总离子流图

### 2.6 实际样品检测

实际样品筛查分为两个步骤顺序进行分析。先直接进行HS-SPME-GC-MS分析,检测完毕再向样品中加入BSTFA,进行衍生化HS-SPME-GC-MS分析。所建立的方法被成功应用于OPCW环境样品水平测试中,涉及样品类型包括泵油、生物柴油、橄榄油等,如第38次水平测试中生物柴油样品385中甲基膦酸二甲酯和386中氯化苦的检测(见图6),第42次水平测试中泵油样品421中二乙烯基硫醚和双(2-氯乙基)二硫化物的检测(见图7a)以及第48次水平测试中橄榄油样品484中硫代膦酸二乙酯和485中甲基膦酸二环己酯等的检测。在处理实际样品时,均取0.1 mL样品用泵油稀释至1 mL(稀释10倍),再按照1.4.1节操作进行样品处理。与OPCW推荐处理方法(耗时>5 h)(见图7b)相比,本方法不仅样品用量少,检测灵敏度高,还省时省力,大大缩短了样品的处理时间(<1 h),在快速筛查方面极具优势。

**图6:**
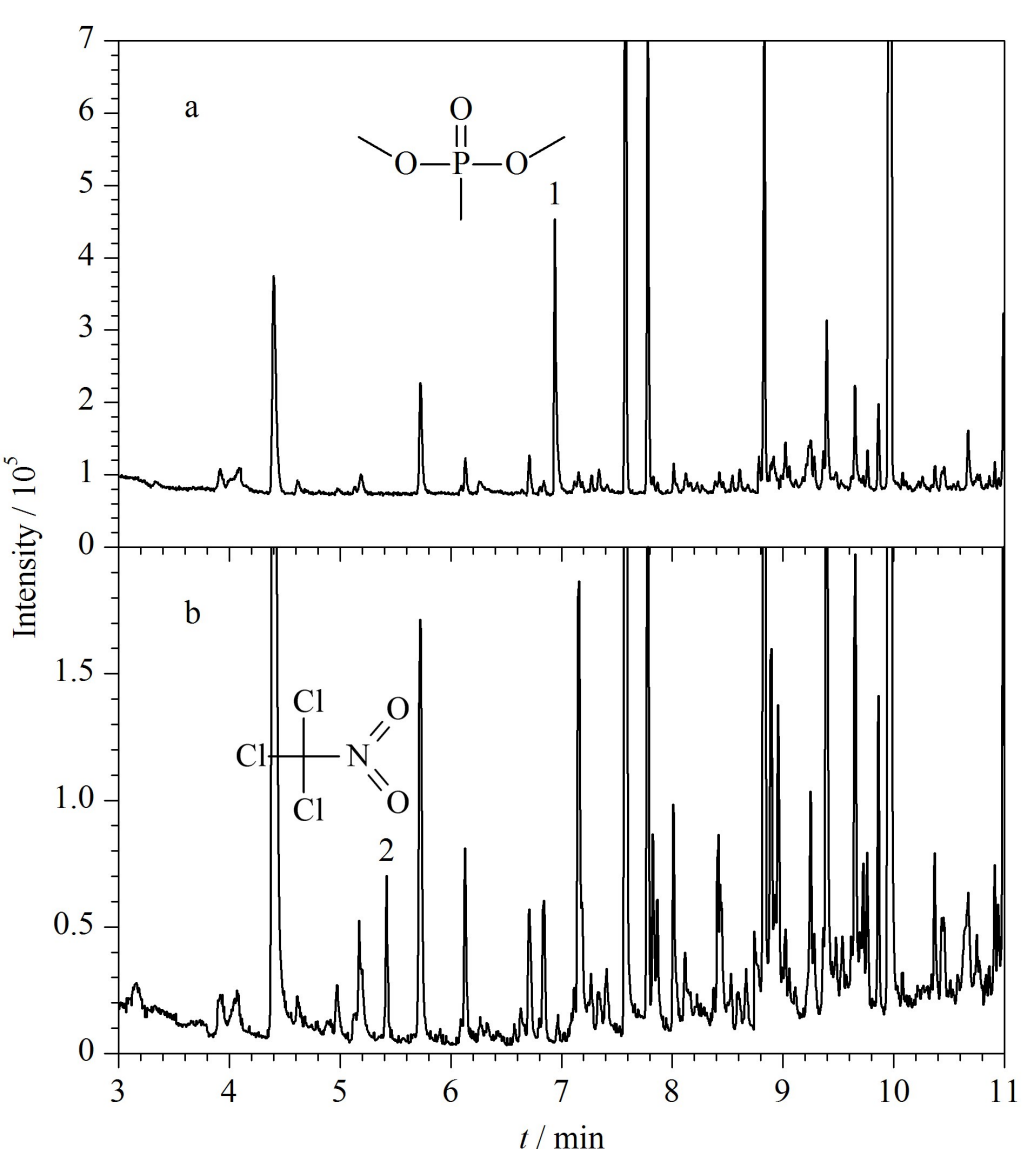
OPCW第38次水平测试中生物柴油基质样品的HS-SPME-GC-MS检测总离子流图

**图7:**
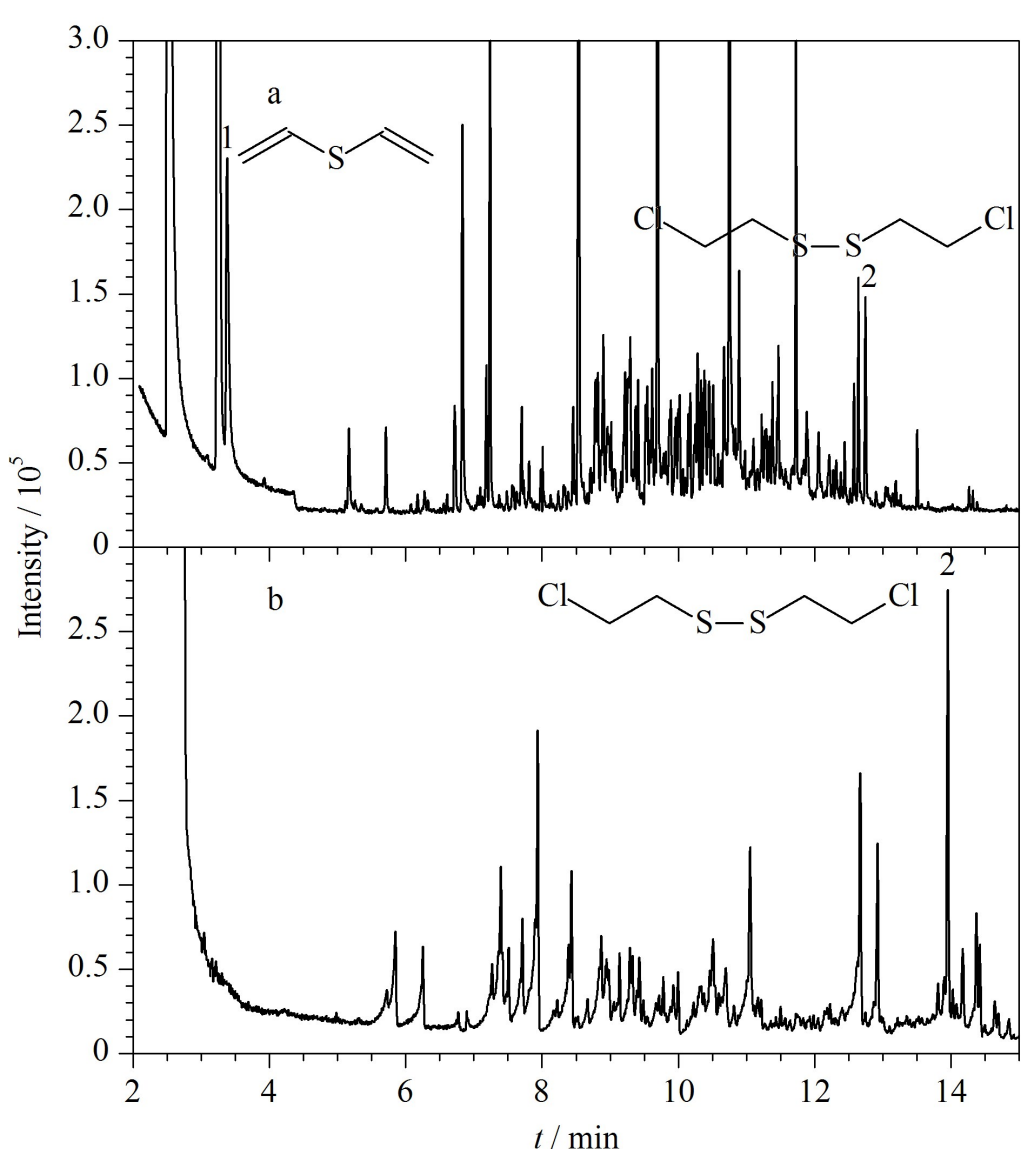
采用不同方法分析OPCW第42次水平测试泵油样品421的GC-MS总离子流图

## 3 结论

本文建立了HS-SPME-GC-MS快速筛查油性基质中挥发性化学武器及其相关化合物的方法,并将其成功应用于OPCW环境样品水平测试中多种油性基质样品的检测分析。与OPCW推荐的净化处理方法相比,采用HS-SPME不仅样品用量少,还将油性基质样品的处理时间从5 h以上缩短至1 h以内,节省了分析时间,特别是可避免样品浓缩导致的低沸点化合物的损失和漏检。该方法可用于检测绝大部分的公约相关化合物,但少数极高沸点的化合物如失能剂二苯羟乙基喹咛酯(BZ)等,由于很难从基质中挥发出来,故不适合采用HS-SPME,仍需采用常规方法进行提取分析。此外,还有少数化合物不适合使用BSTFA进行衍生化分析,如路易氏剂和氨基磺酸类化合物等,但通过更换其他的衍生化试剂仍可实现采用HS-SPME-GC-MS分析,在后续工作中我们将进一步对方法进行完善。

化学武器核查分析是对公约相关化合物的全面筛查和鉴定,其结果具有重大国际影响。为了保证分析结果的准确性,避免漏检误检,有必要将多种筛查手段和分析方法联合使用,互相补充和印证。本文所建立的方法快速、灵敏、简便,重复性好,并具有普适性,可作为常规推荐分析方法的重要补充用于复杂油性基质化学武器公约相关化合物的快速筛查与鉴定。
